# Effect of vitamin D supplementation on upper and lower limb muscle strength and muscle power in athletes: A meta-analysis

**DOI:** 10.1371/journal.pone.0215826

**Published:** 2019-04-30

**Authors:** Lin Zhang, Minghui Quan, Zhen-Bo Cao

**Affiliations:** School of Kinesiology, Shanghai University of Sport, Shanghai, China; Nottingham Trent University, UNITED KINGDOM

## Abstract

**Background:**

Vitamin D may play a role in skeletal muscle because of the discovery of VDR in skeletal muscle. However, vitamin D deficiency is a global problem, including athletes. Studies examining the effect of vitamin D supplementation on muscle function in athletes have inconsistent results. Therefore, we aimed to quantitatively summarize the evidence for the effect of vitamin D supplementation on skeletal muscle strength and explosive power of athletes using a meta-analysis.

**Methods:**

PubMed, EMBASE, Cochrane Library, and Web of Science were searched for studies to identify randomized controlled trials or controlled trials meeting the inclusion criteria. By a meta-analysis, effect sizes (standardized mean differences, SMD) with 95% confidence intervals (CI) was calculated to compare reported outcomes across studies, *I*^*2*^ index was used to assessing heterogeneity, and heterogeneity factors were identified by regression analysis. The potential publication and sensitivity analyses were also assessed.

**Results:**

Eight RCTs involving 284 athletes were included. The protocols used to evaluate the muscle strength of athletes were inconsistent across the included studies, and muscle explosive power was assessed via vertical jump tests. The results indicated that vitamin D supplementation had no impact on overall muscle strength outcomes (SMD 0.05, 95% CI: -0.39 to 0.48, *p* = 0.84). In subgroup analysis, vitamin D supplementation had an effect on lower-limb muscle strength (SMD 0.55, 95% CI:0.12 to 0.98, *p* = 0.01) but not upper-limb muscle strength (SMD -0.19, 95% CI:-0.73 to 0.36, *p* = 0.50) or muscle explosive power (SMD 0.05, 95% CI:-0.24 to 0.34, *p* = 0.73). Vitamin D supplementation was more effective for athletes trained indoors (SMD 0.48, 95% CI:0.06 to 0.90, *p* = 0.02).

**Conclusions:**

Vitamin D supplementation positively affected lower limb muscle strength in athletes, but not upper limb muscle strength or muscle power. Different muscle groups and functions may respond differently to vitamin D supplementation. Additional studies should focus on determining the appropriate vitamin D supplementation methods and optimal serum 25(OH)D levels for athletes.

**Registration:**

The protocol for our study is registered in the international prospective register of systematic reviews (PROSPERO registration number CRD42016045872).

## Introduction

Better muscle function, including muscle strength and power, are crucial factors for athletes, not only as the first step to maintaining excellent performance, but also for the indispensable capacity to decrease sports injury risks [1, 2].

Vitamin D, a fat-soluble sterol compound and hormone precursor, has been suggested to play an important role in skeletal muscle function and metabolism by a number of evidences [3–7]. Vitamin D deficiency, as assessed based on serum 25-hydroxyvitamin D [25(OH)D] concentration, has been associated with impaired muscle action, including diffuse and nonspecific musculoskeletal pain [8], muscle weakness in the elderly [9], sarcopenia development [10, 11], and decreased muscle strength [12]. Several scientific societies have proposed various recommendations for vitamin D insufficiency or deficiency based on serum 25(OH)D concentration [13, 14]. In this study, we used the definition that vitamin D sufficiency as 25(OH)D concentration above 75 nmol/L (30 ng/mL), vitamin D insufficiency from 50 to 75 nmol/L (20–30 ng/mL) and vitamin D deficiency below 50 nmol/L (20 ng/mL). Vitamin D insufficiency and deficiency is very common in athletes. A recent meta-analysis found that 44–67% of athletes had inadequate 25(OH)D concentration [15], which may decrease skeletal muscle function, athletic performance [16, 17], and recovery after training [18], and increase the incidence of muscle injury [19, 20].

Exogenous vitamin D supplementation is considered to be an effective means of improving vitamin D status [21], but the reported effects of vitamin D supplementation on muscle function in athletes have been inconsistent. Although a previous qualitative systematic review suggested positive effects of vitamin D supplementation on muscle function in athletes [22], the magnitude of these effects have not been systematically investigated. Therefore, the aim of this study was to quantitatively summarize the current literature to assess the effect of vitamin D supplementation on muscle strength and power in professional and college athletes.

## Materials and methods

The protocol for our study is registered in the international prospective register of systematic reviews (PROSPERO registration number CRD42016045872).

### Search strategy

In accordance with the Preferred Reporting Items for Systematic Reviews and Meta-analysis (PRISMA) [23] statement (**S1 Table**), the relevant randomized controlled trials (RCTs) and controlled trials published in English and focused on the effect of vitamin D on muscle strength or power were identified by searching PubMed, EMBASE, Cochrane Library, and Web of Science from the start of indexing to October 17, 2017. The following Medical Subject Headings (MeSH) and subject terms or key words were used in the search strategy (Details in **S1 Appendix**): Vitamin D, 25(OH)D, cholecalciferol, 25-hydroxyvitamin D, vitamin D supplementation, muscle, muscle function, muscle strength, muscle power, muscle performance, athletes, player, sportsman, sportswoman.

### Study selection

Two authors (L.Z. and M.Q.) independently screened the literature and identified relevant studies. Any disagreements were resolved by discussion with the third author (Z.C.).

According to the PICO approach, reports were included in this study if they met all of the following criteria: 1) Population (P) consisted of active athletes, university team or varsity athletes, with no restrictions on age or sport; 2) Intervention (I) was oral vitamin D supplementation without other supplements, not limited to any dosage or duration; 3) Comparison (C) was placebo; 4) Outcomes (O) were muscle strength or power measured at baseline and at the termination of intervention for both groups. Only randomized controlled trials were included. Studies were excluded if they were animal trials or involved amateurs and athletes with any disability, chronic illness, or injury.

### Assessment of the methodological quality of studies

The Physiotherapy Evidence Database (PEDro) scale (http://www.pedro.org.au), which includes an 11-item checklist, was used to assess the quality of the included studies [24]. Based on their PEDro scores, the studies were graded as excellent (score 9–10), good (score 6–8), fair (score 4–5), or poor quality (score less than 4).

### Data extraction

The following data were extracted from each included study: author; publication year; number of participants and proportion of females; age; subject description; geographical location, latitude, and season of the supplementation trial; baseline and endpoint 25(OH)D level; duration of intervention; type and dose of vitamin D supplementation; and outcome measures. Subgroup analysis was predetermined based on those data.

### Statistical analysis

All analyses were conducted using STATA statistical software (version 14.0; STATA Corporation LP, College Station, TX). The change in means and standard deviation of muscle strength or muscle power between vitamin D and placebo was used to calculate effect sizes (standardized mean differences, SMD) with 95% confidence intervals (CI) to compare reported outcomes across studies by a meta-analysis. Effect sizes provide a measure of the impact of vitamin D supplementation on muscle strength and muscle power. Conventionally, the effect size is reported as: 0.8, large; 0.5, moderate; and 0.2, small [25].

Between-study heterogeneity was assessed by calculating *I*^*2*^ index. The *I*^*2*^ statistic was used to quantify total variation across studies attributed to heterogeneity rather than sampling error (*I*^2^>50% used as a threshold for significant heterogeneity). Regression analysis was used to identify heterogeneity factors. We measured the potential publication bias by using Egger’s adjusted rank correlation [26]. Sensitivity analyses were conducted by removing one primary study each time to evaluate the quality and consistency of the results [27].

## Results

### Literature search and quality assessment

**Fig 1:**
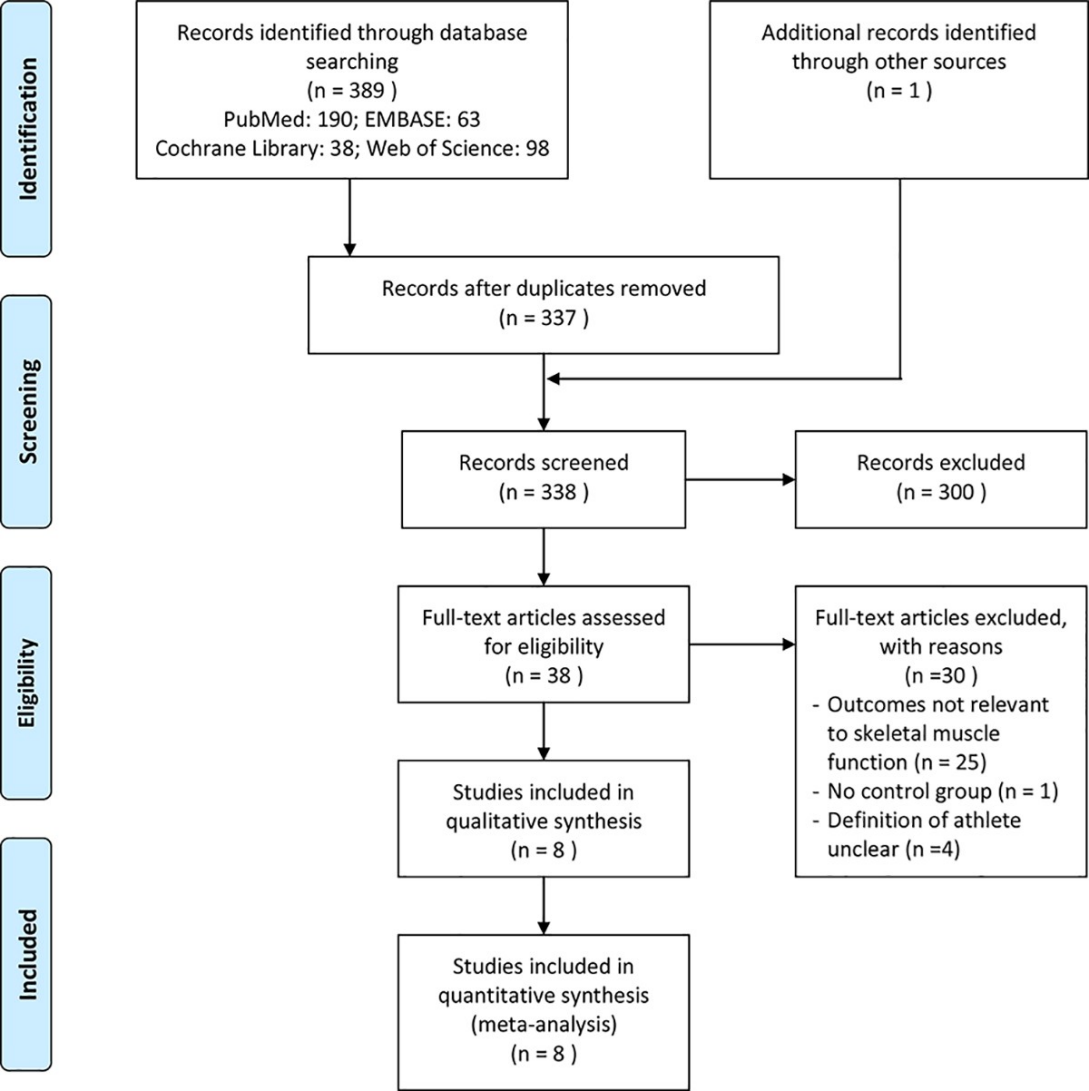
PRISMA 2009 flow diagram for the literature selection process.

A total of 389 records were obtained from PubMed, EMBASE, the Cochrane Library, and Web of Science. After screening, eight RCTs meeting the inclusion criteria were included in the review [16, 17, 28–33]. The flowchart of the search strategy is depicted in **Fig 1**.

By assessment of each study with respect to the criteria of the PEDro scale, the median PEDro score was 9 of 10 (range 7–10). Six studies were considered excellent, and two good (Details in **S2 Table**).

### Study characteristics

A total of 284 subjects were involved in the 8 studies. **Table 1** lists the characteristics of the included studies.

**Table 1:** Summary of included randomized controlled studies in athletes.

Source	*N* (male%)	Age (years)^a^	Location, latitude and season	Baseline 25(OH)D (nmol/L) ^a^	Endpoint 25(OH)D (nmol/L) ^a^	Dose protocol (IU/day)	Study duration, (months)	Outcome(unit)
**Close** **2013a[16]**	10(100)	18±5	UK 53°N Winter	TRE:29±25 ^b^ CON:53±29 70%<50	TRE:103±25 CON:74±24 60% in TRE>100	D_3_ 5000 vs. placebo	2	Bench press 1RM(kg), back squat 1RM(kg), vertical jump (cm)
**Close** **2013b [17]**	30(100)	21.3±1.3	UK 53°N Winter	TRE1:53±26 TRE2:51±26 CON:52±27 57%<50	TRE1:85±10 TRE2:91±24 CON:41±22	D_3_ 20,000IU/ once/ week vs. D_3_ 40,000IU/once/ week vs. placebo	3	Bench press 1RM (kg), leg press 1RM (kg), vertical jump (cm)
**Shanely** **2014[28]**	34(100)	16.2±1.1	USA 35.5°N Winter	TRE:61.1±2.8 CON:62.5±5.6 100%<75 39.4%<50	TRE:66.7±2.7 CON:61.1±5.6	D_2_ 600 vs. placebo	1.5	vertical jump(watts)
**Dubnov-Raz** **2015[29]**	53(57)	14±1.7	Israel 32.4°N~31.6°N Autumn and winter	TRE:61±12.3 CON: 62±11.5 98%<75	TRE:74.0±16.3 CON:50.8±10.5 48% in TRE>75	D_3_ 2000 vs. placebo	3	Handgrip strength (kg)
**Jastrzebska** **2016[30]**	36(100)	17.5±0.6	Poland 54.2°N Winter	TRE:48.5±8.6 CON:47.5±16.2 61.1%<50	TRE:106.3±10.2 CON:43.5±14.5 45% in TRE≥100	D_3_ 5000 vs. placebo	2	Squat jump (cm), countermovement jump (cm)
**Todd** **2016[31]**	42(43)	20±2	UK 55°N Autumn and winter	TRE:47.37±13.3 CON:43.1±22.0 75%<50	TRE:83.68±32.98 CON:49.22±25.40 100% in TRE>50	D_3_ 3000 vs. placebo	3	left and right handgrip strength (kg), countermovement vertical jump (cm)
**Wyon** **2016[32]**	22(100)	27.5±9	UK 52.3°N Winter	TRE:32.9±9.4 ^b^ CON:40.8±6.8 82%<75	TRE:41.9±8.03 CON:40.8±6.4	D_3_ 150,000IU once vs. placebo	8 days	Isokinetic (30°/s) concentric quadriceps and hamstring strength (Nm)
**Fairbairn 2017[33]**	57(100)	21.4±2.8	New Zealand 45–46.5°S Autumn	TRE:93±19 CON: 95±17	TRE:114±19 CON:80±21	D_3_ 50,000IU once a fortnight vs. placebo	3	Bench pull 1RM (kg), Weighted reverse-grip Chin-up 1RM (kg), Bench press 1RM (kg)

^**a**^ Values are presented as mean ± standard deviation (SD), range, median or unless otherwise specified.

^**b**^ Treatment group significantly lower than control group (*p*<0.05).

IU/day: international unit per day. IU: international unit. CON: control group. TRE: treatment group with vitamin D supplementation. TRE1: treatment group with oral vitamin D_3_ 20,000 IU/week for 12 weeks. TRE2: treatment group with oral vitamin D_3_ 40,000 IU/week for 12 weeks. RM: one-repetition maximum.

Six studies involving 214 subjects reported upper or lower limb muscle strength measures [16, 17, 29, 31–33], and five studies involving 152 participants reported the assessment of muscle power [16, 17, 28, 30, 31]. The average age of participants in the 8 studies was 19 years old. Sporting activities that subjects engaged in included soccer [16], football [17, 28, 30, 31], rugby [17, 33], swimming [29], and judoka [32]. Athletes in five studies were professional players and performed prescribed special training [16, 29, 30, 32, 33]; others included university sports athletes [17, 28, 31]. Training experience, reported only in two studies, was 4.4 [29] and 5.5 years [33] respectively. All included studies reported the season that the intervention trial was performed. Five studies were conducted during the winter [16, 17, 28, 30, 32], and three during autumn and winter [29, 31, 33]. The geographic latitude at which data collection occurred was reported in six studies [16, 17, 29, 31–33]; latitudes in the other two studies were inferred from the geographical location provided by the authors [28, 30].

Participants had mean baseline 25(OH)D levels ≥75 nmol/L in one study [33], 50–75 nmol/L in three studies [17, 28, 29], and <50 nmol/L in four studies [16, 30–32]. In one study, all subjects’ baseline 25(OH)D level was <75 nmol/L [28]. Seven studies [16, 17, 29–33] administered vitamin D_3_ as an intervention, and the other one [28] used vitamin D_2_. Supplement dosages ranged from 600 international units (IU) per day to 40,000 IU per week (equivalent to 5714 IU per day) over 6–12 weeks, or 150,000 IU once.

At the end of the intervention, the vitamin D-treated group achieved mean 25(OH)D levels ≥100 nmol/L in three studies [16, 30, 33], 75–100 nmol/L in two studies [17, 31], 50–75 nmol/L in two studies [28, 29], and <50 nmol/L in one study [32].

The protocols used to evaluate the muscle strength of participants were inconsistent across the studies included in this review. To assess upper limb muscle strength, handgrip strength was measured in two studies [29, 31]. One repetition maximum (1 RM) bench press was used in three studies [16, 17, 33]. The study of Fairbairn et al. also used 1RM bench pull and 1RM weighted reverse-grip chin-up [33]. Lower limb muscle strength was assessed by 1 RM back squat in one study [16] and 1 RM leg press in one study [17]. Thigh muscle strength was measured in one study via isokinetic (30°/s) concentric quadriceps and hamstring peak torque [32]. Muscle explosive power was assessed via vertical jump tests, including vertical jump [16, 17, 28], squat jump [30], and countermovement jump [30, 31].

### Meta-analysis

**Fig 2:**
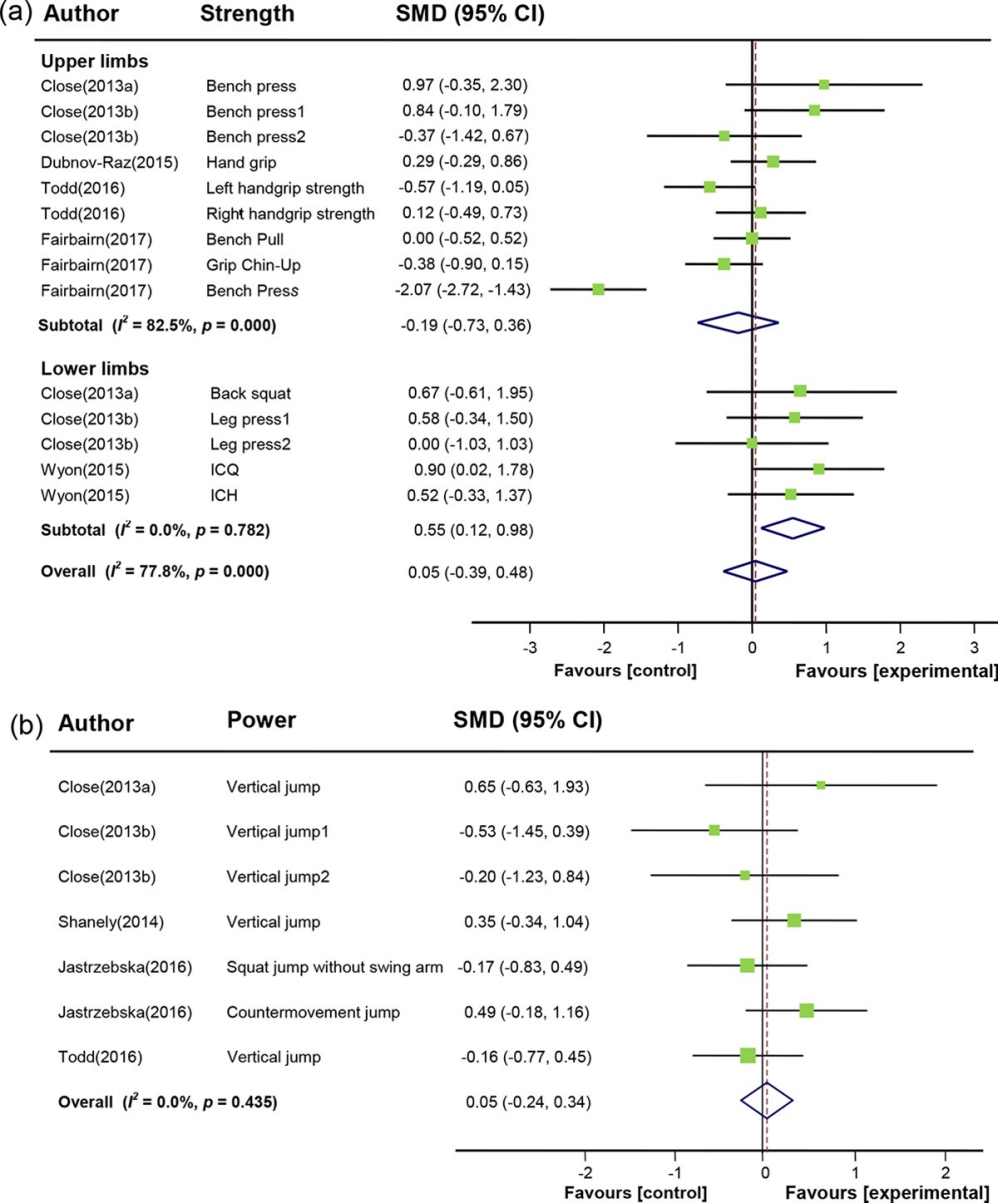
Forest plot displaying the effect of vitamin D supplementation on muscle strength and power. (a) Effect of vitamin D supplementation on upper and lower limb muscle strength. (b) Effect of vitamin D supplementation on muscle power. SMD: standardized mean differences. 95% CI: 95% confidence interval. *I*^*2*^: I-squared test of heterogeneity. Bench press1 / Leg press 1: oral vitamin D_3_ 20000 IU/week for 12 weeks. Bench press 2/ Leg press 2: oral vitamin D_3_ 40000 IU/week for 12 weeks. ICQ: isokinetic concentric quadriceps peak torque. ICH: isokinetic concentric hamstring peak torque. Vertical jump1: oral vitamin D_3_ 20000 IU/week for 12 weeks. Vertical jump2: oral vitamin D_3_ 40000 IU/week for 12 weeks.

Group comparison consisted of six studies was used to assess the effect of vitamin D supplementation on muscle strength (**Fig 2A**). High heterogeneity was present among studies investigating upper limb muscle strength (*I*^*2*^ = 82.5%, *p* = 0.00), with no heterogeneity in those reporting lower limb muscle strength (*I*^*2*^ = 0.0%, *p* = 0.78). Thus, effect sizes were calculated using a random-effects mode. The meta-analysis results indicated that vitamin D supplementation had no impact on overall muscle strength outcomes (SMD 0.05, 95% CI: -0.39 to 0.48, *p* = 0.84). In the subgroup analysis, vitamin D supplementation had a moderate effect (SMD 0.55, 95% CI: 0.12 to 0.98, *p* = 0.01) on increasing lower limb muscle strength in athletes, compared with placebo intake; but no statistically significant effect on upper limb muscle strength (SMD -0.19, 95% CI: -0.73 to 0.36, *p* = 0.50).

Meta-regression analysis for heterogeneity among studies with upper limbs indicated that baseline 25(OH)D concentrations and change of 25(OH)D concentrations in the treatment group were the main factors leading to high heterogeneity (**Table 2**).

**Table 2:** Regression analysis of heterogeneity factors for upper limb muscle strength.

	Coef.	Std. Err.	*t*	*p*>|t|	[95% Conf. Interval]	
**Measure**	.57	.88	0.65	0.58	-3.20	4.34
**Baseline**	-15.55	3.28	-4.74	0.04	-29.66	-1.44
**Endpoint**	-3	2.68	-1.12	0.38	-14.52	8.52
**Change**	-13.26	2.27	-5.86	0.03	-23.0	-3.53
**Duration**	-9.97	3.65	-2.73	0.11	-25.67	5.74
**Environment**	1.23	1.24	0.99	0.43	-4.10	6.57
**_cons**	62.73	8.54	7.34	0.02	25.98	99.49

Coef.: regression coefficients. Std. Err.: the standard error of the regression coefficient. 95% Conf. Interval: 95% confidence interval. In first column, Measure indicates regression coefficients;. Baseline indicates baseline 25(OH)D concentrations;. Endpoint indicates endpoint 25(OH)D concentrations in treatment group;. Change indicates change of 25(OH)D concentrations in treatment group during intervention; Duration indicates study duration; Environment indicates training environment(outdoors, indoors or combination).

In the subgroup analysis, high heterogeneity was shown in those reporting athletes training environment (**Table 3**).

**Table 3:** Subgroup analysis of muscle strength based on participants’ characteristics.

Potential modifiers	No. of studies ^a^	SMD (95% CI)	Heterogeneity
**All studies**	6	0.05(-0.39, 0.48)	*I*^2^ = 77.8%, *p* = 0.00
**Number of athletes**	284		
**Muscle location**			
Upper limbs	5	-0.19 (-0.73, 0.36)	*I*^2^ = 82.5%, *p* = 0.00
Lower limbs	3	0.55 (0.12, 0.98)	*I*^2^ = 0.0%, *p* = 0.78
**Baseline 25(OH)D concentrations (nmol/L)**			
**<75**	5	0.28 (-0.03, 0.59)	*I*^2^ = 31.4%, *p* = 0.15
**≥75**	1	-0.80 (-1.96, 0.36)	*I*^2^ = 92.2%, *p* = 0.00
**Endpoint 25(OH)D concentrations in treatment group (nmol/L)**			
**<90**	4	0.31 (-0.08, 0.69)	*I*^2^ = 47.6%, *p* = 0.08
**≥90**	3	-0.26 (-0.99, 0.48)	*I*^2^ = 82.9%, *p* = 0.00
**Change of 25(OH)D concentrations in treatment group (nmol/L)**			
**<32**	3	-0.15(-0.92, 0.63)	*I*^2^ = 88.8%, *p* = 0.00
**≥32**	3	0.17(-0.23, 0.58)	*I*^2^ = 37.3%, *p* = 0.13
**Vitamin D dosage (IU/day)**			
**<3500**	3	0.18 (-0.28, 0.63)	*I*^2^ = 51.8%, *p* = 0.08
**≥3500**	4	-0.04 (-0.69, 0.62)	*I*^2^ = 82.9%, *p* = 0.00
**Training environment**			
**outdoors**	2	0.11 (-053, 0.75)	*I*^2^ = 53.8%, *p* = 0.09
**indoors**	2	0.48 (0.06, 0.90)	*I*^2^ = 0.0%, *p* = 0.51
**Combination of outdoors and indoors**	2	0.23(-0.95, 0.49)	*I*^2^ = 84.8%, *p* = 0.00
**Study duration (months)**			
**<3**	2	0.74 (0.23, 1.25)	*I*^2^ = 0.0%, *p* = 0.92
**= 3**	4	-0.18 (-0.68, 0.31)	*I*^2^ = 80.4%, *p* = 0.00
**Latitude(°N)**			
**<53**	3	-0.15 (-0.92, 0.63)	*I*^2^ = 88.8%, *p* = 0.00
**≥53**	3	0.17 (-0.23, 0.58)	*I*^2^ = 37.3%, *p* = 0.13

^a^ More than one muscle strength test or vitamin D supplementation dosage may exist in the same study. Not all studies contributed to all strata, so the total n does not always = 6.

SMD: standardized mean differences. CI: confidence interval. *I*^2^: I-squared test of heterogeneity.

We used random-effects meta-analysis to evaluate the effect sizes. Vitamin D supplementation had a small effect (SMD 0.48, 95% CI: 0.06 to 0.90, *p* = 0.024) on increasing muscle strength in athletes training indoors, compared with outdoors or combination training. Furthermore, vitamin D supplementation duration less than 3 months demonstrated a statistically significant improvement of muscle strength (SMD 0.74, 95% CI: 0.23 to 1.25, *p* = 0.00), whereas supplementation for 3 months did not (SMD -0.18, 95% CI: -0.68 to 0.31, *p* = 0.47) by random-effects models in the calculation of effect sizes.

Five studies were included in the analysis of the effect of vitamin D supplementation on muscle power. No significant effect of vitamin D supplementation on muscle power was found (SMD 0.05, 95% CI: -0.24 to 0.34, *p* = 0.73; **Fig 2B**), and no heterogeneity was present (*I*^*2*^ = 0.0%, *p* = 0.44). The results showed that vitamin D supplementation had no significant effect on muscle power between the analyzed subgroups.

Using Egger’s adjusted rank correlation, no publication bias was observed for muscle strength (*p* = 0.17) or muscle power (*p* = 0.59). Sensitivity analysis revealed that both muscle strength and power outcomes were stable when studies were removed one by one. The sensitivity analysis did not affect the heterogeneity of muscle strength or power outcomes.

## Discussion

The purpose of the present meta-analysis was to evaluate the effect of vitamin D supplementation on upper and lower limb muscle strength and muscle explosive power in professional athletes and college athletes. The results of the included studies demonstrated a significant effect of vitamin D supplementation on lower limb muscle strength in athletes, but not on upper limb muscle strength or muscle power.

There are few meta-analyses of controlled or randomized controlled trials that have discussed the effects of vitamin D supplementation on skeletal muscle fitness using different inclusion criteria, and they report inconsistent conclusions. Farrokhyar et al. [21] found hand grip strength increased significantly after 12 weeks of vitamin D supplementation. Tomlinson et al. [34] observed that vitamin D supplementation improved both upper and lower limb muscle strength. The previous review of Tomlinson et al. contained highly heterogeneous populations, including athletic and non-athletic populations, while our participants were healthy, active athletes with an average age of 19 years, supplemented by vitamin D alone. Our study performed the quantitative meta-analysis using a more consistent study population, and included a larger number of studies (8 RCTs in athletes), and thus, provided a larger sample and more representative results.

Increasing evidence showed that vitamin D exerted important roles in skeletal muscle fitness, and affected muscle function through regulating both calcium-phosphorous homeostasis and related protein transcription [3, 4, 35]. Vitamin D receptor (VDR) and 1,25(OH)_2_D (the active metabolite of vitamin D) have been shown to have a key function in this relationship as proven by several human or animal studies. A significant association between the VDR genotypes and muscle strength was observed in elderly [36], young [37, 38], and mixed populations [3, 39]. However, it cannot be ignored that evidence is limited and some studies failed to observe an association between different polymorphisms and various muscle function measurements [3, 40, 41]. The animal studies also provided insights into this relationship. Girgis et al. found that vitamin D receptor knockout (VDRKO) resulted in a significant decrease in muscle strength and muscle fibers size [42]. Furthermore, the active metabolite of vitamin D, 1,25(OH)_2_D, could regulate mitochondrial function, dynamics, and enzyme function in human skeletal muscle cells [5], which may partly explain the underlying mechanism of the effect of vitamin D on skeletal muscle function from a metabolic point of view. A longitudinal study showed that cholecalciferol therapy for 10–12 weeks augmented muscle mitochondrial maximal oxidative phosphorylation in vitamin D-deficient individuals [7]. This issue should be explored in more depth.

### The response of muscle strength to vitamin D supplementation

The effect of vitamin D supplementation on muscle function was firstly reported in the previous studies of myopathy patients, whose muscle strength improved remarkably after vitamin D treatment [43–45]. Ceglia and Pojednic, et al. found that vitamin D supplementation in older women not only increased muscle fiber size [46], but also the intramyonuclear VDR concentration [46, 47], and a significant increase in VDR percent in type II fibers was further observed [46]. The interaction between VDR and muscle fiber type needs to be explored. It seemed that elderly or low baseline vitamin D population may get more beneficial effect of vitamin D supplementation on muscle function [48–50].

Our observation of a significant effect of vitamin D supplementation on the muscle strength of lower but not upper limbs is consistent with the findings of Cangussu et al.[47]. The mechanisms for the suggested differential effect of vitamin D on upper and lower limb muscle strength remains unclear but may be associated with several factors.

First, VDR expression of different muscle groups may contribute to the differential effects between the upper and lower limb musculature. Previous studies in cell culture and animals have indicated that the content of VDR in target tissues was positively associated with the functional response level of tissues to vitamin D [51–53]. Future studies are needed to explore the observation.

Second, it is possible that upper limb strength measures (e.g. isometric handgrip dynamometry) are less sensitive to modest changes in muscle strength and thus lack sufficient power to detect small but significant strength gains in the upper extremities [54]. Thus, sensitive and standardized measurement techniques for athletes are urgently needed. Moreover, the lower limbs are more frequently utilized for load bearing during daily life and exercise compared with the upper limbs, which may enhance neuromuscular modulation of leg strength by stimuli [55] and increase the capillary density in leg muscle [56]. Therefore, compared to upper limb muscle, lower limb muscle strength may react to vitamin D supplementation more quickly and obviously.

We also found that vitamin D supplementation duration of less than 3 months demonstrated a statistically significant improvement of muscle strength, whereas supplementation for 3 months did not. However, this result should be interpreted with caution. All studies lasting 3 months, included seven upper limb muscle strength tests and one lower limb muscle strength; thus, the merged results may be influenced by the higher number of upper limb muscle strength tests.

### Optimal vitamin D level for muscle function

The optimal vitamin D level for muscle strength is a focus of ongoing research, but currently remains unresolved. The Institute of Medicine (IOM) recommends that vitamin D deficiency is defined as serum 25(OH)D concentration below 50 nmol/L, and vitamin D sufficiency as at least 50 nmol/L and preferably more than 75 nmol/L. However, the IOM report stressed that its recommendations for vitamin D were based primarily on the intake (and serum 25(OH)D concentration) needed to ensure skeletal health [57], while the recommendations serum 25(OH)D concentrations for preserving neuromuscular performance have yet to be established. The optimal levels of 25(OH)D for athletes have not been defined either.

The studies included in our meta-analysis had a wide range of baseline vitamin D concentrations (29–93 nmol/L). Despite a significant increase in vitamin D status being reported in all the included studies, the change in magnitude of muscle strength and muscle power outcomes did not entirely correspond to the increase in concentration. Close et al. [16] reported a significant increase in muscle strength and vertical jump height after vitamin D supplementation. Wyon et al. [32] and Fairbairn et al. [33] also found a significant increase in some of the muscle strength measurements. Other included studies did not observe a significant change in muscle function after vitamin D intervention, although vitamin D concentration was up to 114 nmol/L, and the average change of vitamin D concentration was 32 nmol/L. Some randomized trial in elderly found that although high doses of vitamin D were effective in reaching the threshold of 75 nmol/L of 25(OH)D concentration, it may have deleterious effects on muscle function and the risk of falls [58–60]. It 's also worth noting that supplementation with high doses of vitamin D to subjects without vitamin D deficiency may have no effects [17, 30] or negative effects [61]. Thus, the biochemical safety of vitamin D supplementation for the athletes also need to be considered. And it raised a question as to whether an optimal vitamin D concentration for muscle function existed, especially for athletes. Furthermore, even if it exists, what should it be based on?

The lowest 25(OH)D levels of athletes are typically observed in the winter and early spring [62]. To ensure vitamin D sufficiency in mid-winter, Galan et al. [63] suggested that a serum 25-hydroxyvitamin D concentration of 122.7 nmol/L was required in early autumn to avoid levels falling below 75 nmol/L among professional football players.

The choice of an indoor or outdoor training environment also influences sun exposure and thus ultimately affects vitamin D synthesis. Emerging evidence has indicated that athletes who train outdoors have higher vitamin D levels compared to those who train indoor or avoid peak daylight hours, regardless of latitude or season [64, 65]. Our meta-analysis suggests that vitamin D supplementation may be more effective for athletes who perform indoor training. Moreover, as athletes in weightrestricting sports may be more prone to vitamin D deficiency than others [16], we suggested that their vitamin D status be tested regularly. Altogether, vitamin D deficiency in athletes, especially in athletes training indoors, should be treated with vitamin D supplementation appropriately, in order to achieve an adequate vitamin D concentration. However, it is not recommended that athletes take large amounts of vitamin D for the purpose of improving sports performance or training effect, due to insufficient evidence in this regard.

### Change in muscle power

No significant effect of vitamin D supplementation on muscle power was found, which may indicate that muscle strength and power have different responses to vitamin D supplementation in athletes. Muscle power, for example vertical jump, mainly utilizes fast-twitch fibers (type II), while muscle strength may utilize both type I and type II or type II muscle [66]. Muscle power has been shown to decrease more rapidly than strength in elderly individuals with sarcopenia [67]. Another study on elderly subjects [68] suggested a type II fiber-focused response to vitamin D_3_ supplementation. Some studies have shown that vitamin D deficiency results in proximal muscle weakness with a reduction in type II muscle fiber [35, 66]. While the data on younger athletes with better muscle function are still limited, it is not possible to determine whether the different responses of muscle strength and power in athletes is related to muscle fiber type.

Furthermore, since many athletic events are either defined by or heavily dependent on muscle power, muscle power impacts athletic performance (e.g. sprinting, jumping). Some of the included studies also measured athletic performance. Close et al. [16] found significant improvement in 10 m sprint and vertical jump in the vitamin D supplementation group, whereas the placebo group showed no change. By contrast, other studies showed that increasing serum 25(OH)D had no significant effect on sprint performance [17, 30, 33]. Thus, while vitamin D supplementation may improve muscle strength, the lack of improvement in power suggests that there may not be any direct benefit to athletic performance.

### Strengths and limitations

This report is the first meta-analysis to quantitatively assess the effects of vitamin D supplementation on muscle strength and power in highly trained athletes in RCTs. Sensitivity analysis revealed that our results were stable. However, this study also has some limitations. First, the variety of training programs and sports of included athletes may be a limitation. However, all studies reported the subjects performing specified training plans. Second, the possibility that potentially relevant studies may have been missed due to the limitation of our search to English-language publications in our study cannot be excluded.

## Conclusions

This meta-analysis demonstrated that, while it showed no improvement in muscle power, vitamin D supplementation had a significant effect on lower limb muscle strength, especially in athletes who train indoors. These findings suggest that achieving vitamin D-sufficient status through supplementation of vitamin D is necessary for maintaining greater muscle strength, and there may be a discrepancy between upper and lower limb muscle strength in response to vitamin D supplementation among athletes, especially those who train indoors.

## Supporting information

S1 Table PRISMA 2009 checklist. (DOC)

S2 Table Quality scores for eligible studies. PEDro Physiotherapy Evidence Database, + the item was clearly satisfied. The PEDro scale is based on the Delphi list developed by Verhage et al. at the Department of Epidemiology, University of Maastricht.[69] Only criteria 2–11 are scored, for a maximum total of 10: 1 eligibility criteria, 2 randomization, 3 concealed allocation, 4 groups similar at baseline, 5 blinding subjects, 6 blinding therapists, 7 blinding assessors, 8 measures obtained for > 85%, 9 intention to treat, 10 between-group statistical comparison, 11 point measures of variability.
^a^ Column 1 not used in the calculation of the scores.
(DOCX)

S1 Appendix Detailed search strategies. (DOCX)
