# Supplementary material for: Effect of vitamin D supplementation on upper and lower limb muscle strength and muscle power in athletes: A meta-analysis
Source: PLoS One. 2019 Apr 30;14(4):e0215826. doi: 10.1371/journal.pone.0215826 (PMC6490896; doi:10.1371/journal.pone.0215826)
Supplement: S2 Table — PEDro Physiotherapy Evidence Database, + the item was clearly satisfied. The PEDro scale is based on the Delphi list developed by Verhage et al. at the Department of Epidemiology, University of Maastricht.[69] Only criteria 2–11 are scored, for a maximum total of 10: 1 eligibility criteria, 2 randomization, 3 concealed allocation, 4 groups similar at baseline, 5 blinding subjects, 6 blinding therapists, 7 blinding assessors, 8 measures obtained for > 85%, 9 intention to treat, 10 between-group statistical comparison, 11 point measures of variability. a Column 1 not used in the calculation of the scores. (DOCX) [file pone.0215826.s002.docx]

S1 Table. Quality scores for eligible studies.

| **Author** | **PEDro scale items** | | | | | | | | | | | **Total** |
| --- | --- | --- | --- | --- | --- | --- | --- | --- | --- | --- | --- | --- |
|  | **1^a^** | **2** | **3** | **4** | **5** | **6** | **7** | **8** | **9** | **10** | **11** |  |
| **Close et al.[**[**14**](#_ENREF_14)**]** | + | + | + | + | + | + | + |  | + | + | + | 9 |
| **Close et al.[**[**15**](#_ENREF_15)**]** | + | + | + | + | + | + | + |  | + | + | + | 9 |
| **Shanely et al.[**[**26**](#_ENREF_26)**]** | + | + | + | + | + | + | + | + | + | + | + | 10 |
| **Dubnov-Raz et al.[**[**27**](#_ENREF_27)**]** | + | + | + | + | + | + | + | + | + | + | + | 10 |
| **Jastrzebska et al.[**[**28**](#_ENREF_28)**]** | + |  |  | + | + | + | + |  | + | + | + | 7 |
| **Todd et al.[**[**29**](#_ENREF_29)**]** | + | + | + |  | + | + | + |  | + | + | + | 8 |
| **Wyon et al.[**[**30**](#_ENREF_30)**]** | + | + | + |  | + | + | + | + | + | + | + | 9 |
| **Fairbairn et al.[**[**31**](#_ENREF_31)**]** | + | + | + | + | + | + | + | + | + | + | + | 10 |
| **Total** | 8 | 7 | 7 | 6 | 8 | 8 | 8 | 4 | 8 | 8 | 8 | Median 9 |

PEDro Physiotherapy Evidence Database, + the item was clearly satisfied.

The PEDro scale is based on the Delphi list developed by Verhage et al. at the Department of Epidemiology, University of Maastricht.[[58](#_ENREF_58)] Only criteria 2-11 are scored, for a maximum total of 10: 1 eligibility criteria, 2 randomization, 3 concealed allocation, 4 groups similar at baseline, 5 blinding subjects, 6 blinding therapists, 7 blinding assessors, 8 measures obtained for > 85%, 9 intention to treat, 10 between-group statistical comparison, 11 point measures of variability.

^a^ Column 1 not used in the calculation of the scores.
