# Supplementary material for: Effect of vitamin D supplementation on upper and lower limb muscle strength and muscle power in athletes: A meta-analysis
Source: PLoS One. 2019 Apr 30;14(4):e0215826. doi: 10.1371/journal.pone.0215826 (PMC6490896; doi:10.1371/journal.pone.0215826)
Supplement: S1 Appendix — (DOCX) [file pone.0215826.s003.docx]

S1 Appendix. Detailed search strategies.

To search for studies published in English, the databases included: PubMed, EMBASE, Cochrane Library, and Web of Science from the earliest time to October 17, 2017, using the terms: ("vitamin d"[MeSH Terms] OR "25(OH)D"[All Fields] OR "cholecalciferol"[All Fields] OR "25-hydroxyvitamin D"[All Fields] OR "vitamin D supplementation"[All Fields]) AND ("athletes"[MeSH Terms] OR "athletes"[All Fields] OR "player"[All Fields] OR " sportsman"[All Fields] OR " sportswoman"[All Fields]).
